# Supplementary material for: Availability and Use of Mobile Health Technology for Disease Diagnosis and Treatment Support by Health Workers in the Ashanti Region of Ghana: A Cross-Sectional Survey
Source: Diagnostics (Basel). 2021 Jul 9;11(7):1233. doi: 10.3390/diagnostics11071233 (PMC8307884; doi:10.3390/diagnostics11071233)
Supplement: Supplementary file 1 [file diagnostics-11-01233-s001.zip › diagnostics-1244483-supplementary.pdf]

Supplementary Materials

Table S1. Characteristics of participants from the 100 healthcare facilities surveyed in Ashanti Region.

Variable	Frequency (N = 285)	Percentage (%)
Age		
(20-30)	120	42.11
(31-40)	137	48.07
(41-50)	27	9.47
(51-60)	1	0.35
Sex		
Male	146	51.23
Female	139	48.77
Categories of health professionals		
Medical doctor	44	15.44
Physician assistant	65	22.81
Midwife	7	2.46
General nurse	82	28.7
Community health nurse	26	9.12
Laboratory scientist/technicians	31	10.88
Pharmacist/dispensing technicians	30	10.53

Type of facility	140	49.12
District hospital	101	35.44
Health centre	44	15.44
Clinic		
	**Mean (standard deviation)**	**95% Confidence Interval**
Total number of health professionals	57.8 ± 30.1	20-98
Number of patients per week	175.4 ± 68.4	74-372

Source: Author's computation based on data obtained from the field survey, 2020.

Table S2. Availability of mobile health for diagnostics and treatment support in the Ashanti Region.

**Variable**	**Frequency (N = 285)**	**Percentage (%)**

**Available healthcare infrastructure**		
Mobile wireless devices		
Yes	276	96.84
No	9	3.16
Mobile intervention availability		
Yes	185	64.91
No	100	35.08
Types of mobile health intervention		
Short Message Service		
Yes	149	80.54
No	36	19.46
Phone calls		
Yes	183	98.92
No	2	1.08
Mobile apps		
Yes	43	23.24
No	142	76.76
Multimedia Service		
Yes	2	1.08
No	183	98.92
Video conferencing		

Yes	-	-
No	185	100
Toll free lines		
Yes	37	20.00
No	148	80.00
Types of wireless mobile devices		
Mobile phones		
Yes	185	100.00
No	-	-
Smartphones		
Yes	133	71.89
No	52	28.11
Tablets		
Yes	107	57.84
No	78	42.16
Personal digital assistants		
Yes	-	-
No	185	100.00
Handheld devices		
Yes	-	-
No	185	100.00
Patient monitoring devices		

Yes	2	1.08
No	183	98.92
Watches		
Yes	-	-
No	185	100.00
Continuous supply of power		
Yes	149	80.54
No	36	19.46
Available support systems		
Yes	106	57.30
No	79	42.70
**Healthcare workforce competency**		
Requisite skills for diagnostics		
Yes	132	71.35
No	53	28.65
Competence for treatment		
Yes	164	88.65
No	21	11.35

Not applicable: Frequencies that are not up to the sample size (285) are respondents without access to some mHealth apps.

Source: Author's computation based on data obtained from the field survey, 2020.

Table S3. Use of mobile health for diagnostics and treatment support in the Ashanti Region.

**Variable**	**Frequency (N = 285)**	**Percentage (%)**
**You and your mobile wireless device**		
Have you ever used or currently using mHealth interventions		
Yes	182	98.38
No	3	1.62
Type of diseases mHealth has been used for		
HIV		
Yes	177	95.68
No	8	4.32
TB		
Yes	171	92.43
No	14	7.57
Hypertension		
Yes	99	53.51
No	86	46.49
Diabetes		
Yes	79	42.70
No	106	57.30

Stroke		
Yes	-	-
No	185	100.00
Cancer		
Yes	5	2.70
No	180	97.30
Chronic Respiratory Disease		
Yes	2	1.08
No	183	98.92
Malaria		
Yes	93	50.54
No	91	49.46
Diarrhoea		
Yes	17	9.19
No	168	90.81
Have you ever used smart mobile wireless device		
Health/medical information		
Yes	117	63.24
No	68	36.76
Disease diagnosis		
Yes	182	98.38
No	3	1.62

Treat and manage disease conditions		
Yes	162	87.57
No	23	12.43
Treat and monitor patients' health conditions		
Yes	144	77.84
No	41	22.16
How often are mobile wireless device used for diagnostic purposes		
Once a month	48	25.95
2 or 3 times a month	48	25.95
1 to 6 times a week	57	30.81
Once a day or more	32	17.30
How often are mobile wireless device used for treatment, monitoring, and management of diseases		
Once a month	58	31.35
2 or 3 times a month	48	25.95
1 to 6 times a week	44	23.78
Once a day or more	35	18.92
**Usefulness of mHealth interventions**		
For monitoring patients' disease conditions		
Yes	248	87.63

No	35	12.37
For managing non-communicable diseases		
Yes	189	66.32
No	96	33.68
For managing communicable diseases		
Yes	239	83.86
No	46	16.14
Reminders for treatment procedures adherence		
Yes	175	61.40
No	110	38.60
Reminders for patients' medication adherence		
Yes	234	82.11
No	51	17.89
Reminders to honour clinic appointments		
Yes	229	80.35
No	56	19.65
Reminders for collection of ART and other drugs on time		
Yes	247	86.67
No	38	13.33
For follow-ups to promote treatment compliance		
Yes	210	73.68
No	75	26.32

For supporting patients' test result notifications		
Yes	187	65.61
No	98	34.39
For improving treatment and management of disease conditions		
Yes	213	74.74
No	72	25.26
For making accurate diagnostic decisions		
Yes	253	88.77
No	32	11.23
To increase the effectiveness of treatment and management of diseases		
Yes	249	87.37
No	36	12.63
**Ease of use**		
Easy to use mHealth to support disease diagnosis		
Yes	262	91.93
No	23	8.07
Easy to use mHealth to support the treatment of patients' disease conditions		
Yes	273	95.79
No	12	4.21
Flexible to interact with mHealth interventions		

Yes	273	95.79
No	12	4.21
Frustrating to interact with mHealth applications		
Yes	9	3.16
No	276	96.84
Easy to become skillful in using mHealth		
Yes	267	93.68
No	18	6.32
Easy to learn how to use mHealth devices for diagnosis and treatment		
Yes	267	93.68
**No**	18	6.32
**User satisfaction**		
Comfortable in using mHealth		
Yes	266	93.33
No	19	6.67
Confident in using mHealth		
Yes	254	89.12
No	31	10.88
Completely satisfied with using mHealth		
Yes	218	76.49
No	67	23.51

Using mHealth will increase the quality of healthcare delivery		
Yes	268	94.04
No	17	5.96
**Behavioural intention to use**		
Would you use mHealth for the treatment and management of patients' disease conditions		
Yes	254	89.12
No	31	10.88
Would you always use mHealth for disease diagnosis and treatment support		
Yes	224	78.60
No	61	21.40
Intend to use mHealth for disease diagnosis and treatment support		
Yes	279	97.89
No	6	2.11

Not applicable: Frequencies that are not up to the sample size (285) are respondents without access to some mHealth apps.

Source: Author's computation based on data obtained from the field survey, 2020.

Table S4. Chi-square test results of the relationship between the available health infrastructure or healthcare workforce competency and ownership of mobile wireless devices.

**Availability of Health Infrastructure**	**Ownership of mobile wireless devices**																	
	Ever used or currently using mHealth		Malaria		Hypertension		Tuberculosis (TB)		Diabetes		HIV		Cancer		Disease diagnosis		Monitor patients' conditions	
	**Chi-Square**	**p-value**	**Chi-Square**	**p-value**	**Chi-Square**	**p-value**	**Chi-Square**	**p-value**	**Chi-Square**	**p-value**	**Chi-Square**	**p-value**	**Chi-Square**	**p-value**	**Chi-Square**	**p-value**	**Chi-Square**	**p-value**
Availability of mobile wireless devices	10.88	0.001	4.12	0.041	-	-	-	-	-	-	-	-	-	-	-	-	-	-

mHealth intervention availability	-	-	6.34	0.012	-	-	-	-	-	-	-	-	-	-	-	-	-	-
SMS	-	-	-	-	6.29	0.012	-	-	-	-	-	-	-	-	-	-	-	-
Mobile apps	-	-	-	-	-	-	4.59	0.032	11.5	0.001			-	-	10.1	0.002	-	-
Toll-free	-	-	-	-	-	-	4.95	0.026	-	-	9.44	0.002	-	-	-	-		
Smartphones	-	-	-	-	-	-	9.81	0.002	11.4	0.001	9.09	0.003	-	-	-	-	4.65	0.031
Supply of power	4.34	0.037	-	-	-	-	-	-	-	-	-	-	-	-	-	-	7.25	0.007
Support systems	-	-	-	-	-	-	5.11	0.024	-	-	-	-	6.89	0.009	4.09	0.043	-	-
Requisite skills for diagnostic purposes	-	-	-	-	-	-	6.02	0.014	-	-	4.69	0.030	-	-	-	-	-	-
Competence to use mHealth for treatment	-	-	-	-	-	-	8.93	0.003	-	-	-	-	12.1	0.001	-	-	-	-

Source: Author's computation based on data obtained from the field survey, 2020.

Table S5. Chi-square test results of the relationship between the available health infrastructure or healthcare workforce competency and usefulness of mHealth applications.

**Availability of Health Infrastructure**	**Usefulness of mHealth applications**															
	Manage non-communicable diseases		Manage communicable diseases		Reminders for treatment adherence procedures		Reminders for clinic appointments		Reminders for drugs collection		Follow-ups to promote treatment compliance		Treating and managing other disease conditions		Test result notifications	
	**Chi-Square**	**p-value**	**Chi-Square**	**p-value**	**Chi-Square**	**p-value**	**Chi-Square**	**p-value**	**Chi-Square**	**p-value**	**Chi-Square**	**p-value**	**Chi-Square**	**p-value**	**Chi-Square**	**p-value**
Availability of mobile wireless devices	36.99	0.001	8.09	0.004	17.53	0.001	4.46	0.035	7.59	0.006	7.78	0.005	7.76	0.005	-	-
mHealth intervention availability	-	-	-	-	4.14	0.042	8.22	0.004	-	-	10.84	0.001	-	-	4.83	0.028
SMS	4.80	0.028	-	-	-	-	-	-	-	-	3.95	0.047	-	-	-	-
Phone calls	-	-	4.21	0.040	7.57	0.006	6.68	0.010	7.10	0.008	8.67	0.003	-	-	4.56	0.033
Mobile apps	6.00	0.014	-	-	-	-	-	-	-	-	10.05	0.002	-	-	-	-
Toll-free	-	-	-	-	-	-	5.52	0.019	9.06	0.003	14.60	0.001	7.49	0.006	-	-
Supply of power	-	-	6.29	0.012	-	-	-	-	8.61	0.003-	-	-	-	-	-	-

Support systems	14.07	0.001	7.08	0.008	-	-	3.94	0.047	5.28	0.022	-	-	-	-	-	-
Requisite skills for diagnostic purposes	19.54	0.001	9.37	0.002	-	-	-	-	-	-	-	-	-	-	-	-
Competence to use mHealth for treatment	7.12	0.008	6.60	0.010	-	-	-	-	-	-	-	-	-	-	-	-

Source: Author's computation based on data obtained from the field survey, 2020.

Table S6: Chi-square test results of the relationship between the available health infrastructure or healthcare workforce competency and ease of use of mHealth applications.

**Availability of Health Infrastructure**	**Ease of use of mHealth applications**											
	Easy to use mHealth for disease diagnosis		Easy to use mHealth to support treatment		Flexible to interact with mHealth applications		Frustrating to interact with mHealth applications		Easy to become skilful in using mHealth applications		Easy to learn how to use mHealth devices	
	**Chi-Square**	**p-value**	**Chi-Square**	**p-value**	**Chi-Square**	**p-value**	**Chi-Square**	**p-value**	**Chi-Square**	**p-value**	**Chi-Square**	**p-value**
Availability of mobile wireless devices	2.78	0.037	3.82	0.036	2.41	0.052	-	-	-	-	-	-

mHealth intervention availability	-	-	4.20	0.040	4.47	0.035	-	-	-	-	-	-
SMS	-	-	4.08	0.043	5.04	0.025	-	-	-	-	-	-
Phone calls	11.34	0.001	17.66	0.001	31.98	0.001	-	-	11.86	0.001	24.69	0.001
Mobile apps	-	-	-	-	-	-	-	-	2.20	0.013	-	-
Toll-free	-	-	-	-	4.74	0.030	-	-	-	-	-	-
Supply of power	-	-	-	-	-	-	-	-	2.54	0.011	-	-
Support systems	11.13	0.001	3.62	0.050	-	-	-	-	-	-	2.88	0.049
Requisite skills for diagnostic purposes	2.80	0.036	5.96	0.015	-	-	-	-	2.73	0.032	-	-
Competence to use mHealth for treatment	3.33	0.050	-	-	1.49	0.021	-	-	2.06	0.003	-	-

Source: Author's computation based on data obtained from the field survey, 2020.

Table S7. Chi-square test results of the relationship between the available health infrastructure or healthcare workforce competency and user satisfaction and behavioural intention to use mHealth.

**Availability of Health Infrastructure**	**User satisfaction of mHealth applications**								**Behavioural intention to use mHealth applications**					
	Comfortable using mHealth		Confident in using mHealth		Completely satisfied with mHealth		mHealth can increase quality healthcare		Able to use mHealth to treat and manage patients' conditions		Always use mHealth for disease diagnosis and treatment support		Intend to use mHealth for disease diagnosis and treatment support	
	**Chi-Square**	**p-value**	**Chi-Square**	**p-value**	**Chi-Square**	**p-value**	**Chi-Square**	**p-value**	**Chi-Square**	**p-value**	**Chi-Square**	**p-value**	**Chi-Square**	**p-value**
Availability of mobile wireless devices	-	-	3.61	0.053	4.83	0.028	-	-	-	-	1.23	0.026	-	-
mHealth intervention availability	1.84	0.015	1.03	0.031	-	-	1.39	0.023	-	-	3.18	0.045	-	-
SMS	-	-	-	-	3.27	0.051	-	-	1.14	0.028	-	-	-	-
Phone calls	5.20	0.023	-	-	-	-	7.10	0.008	-	-	-	-	7.57	0.006
Mobile apps	-	-	-	-	2.49	0.011	-	-	-	-	1.78	0.018	-	-
Toll-free	2.69	0.040	-	-	-	-	-	-	1.32	0.025	-	-	-	-
Supply of power	2.55	0.011	2.01	0.015	-	-	-	-	-	-	-	-	1.52	0.046
Support systems	7.96	0.005	-	-	5.56	0.018	-	-	-	-	2.75	0.037	-	-
Requisite skills for diagnostic purposes	6.02	0.014	-	-	-	-	15.9	0.001	4.34	0.037	-	-	-	-
Competence to use	4.46	0.035	-	-	8.89	0.003	-	-	-	-	13.2	0.001	-	-

mHealth for treatment														

Source: Author's computation based on data obtained from the field survey, 2020.

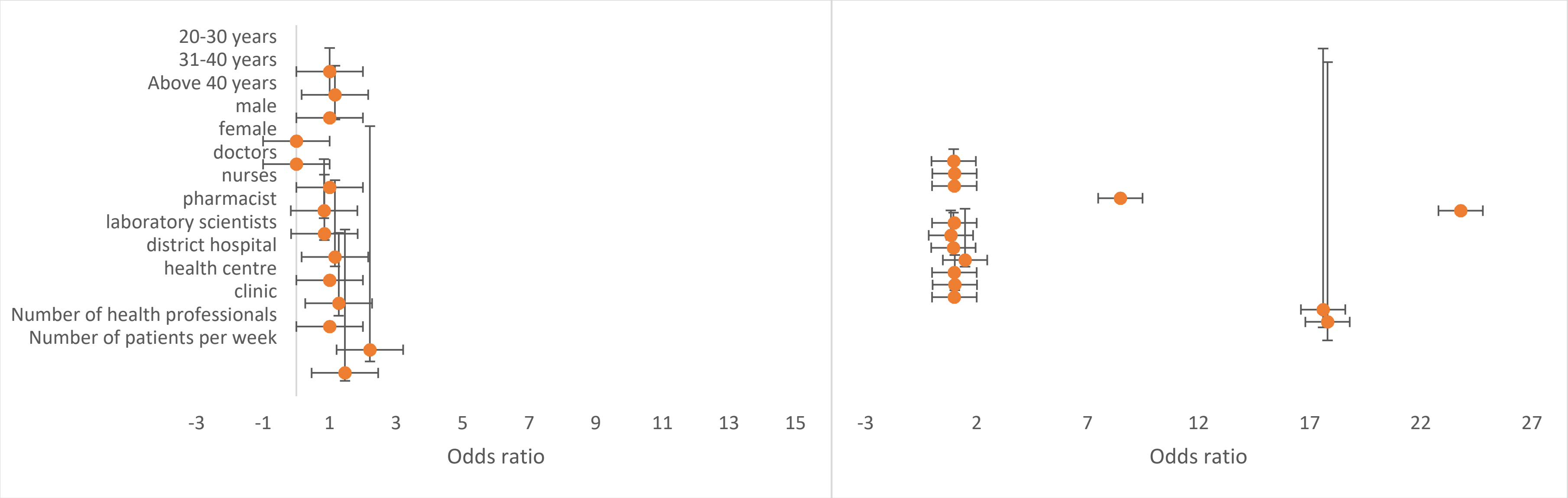

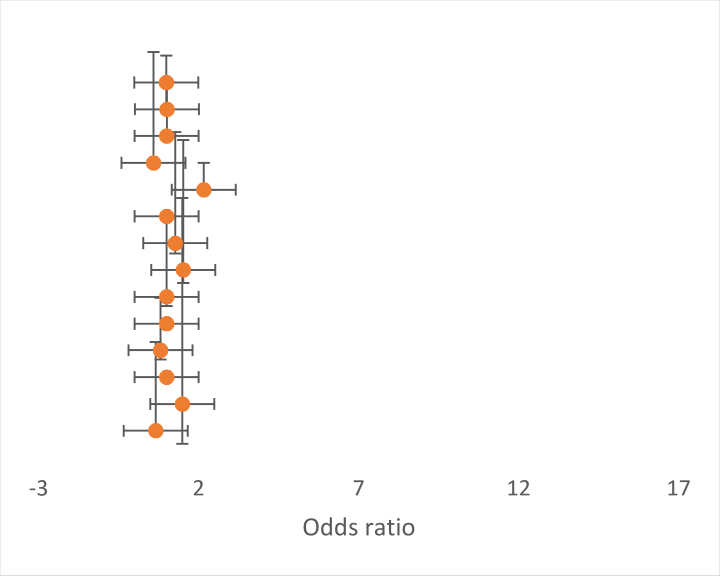
-3
2
7
12
17
Odds ratio

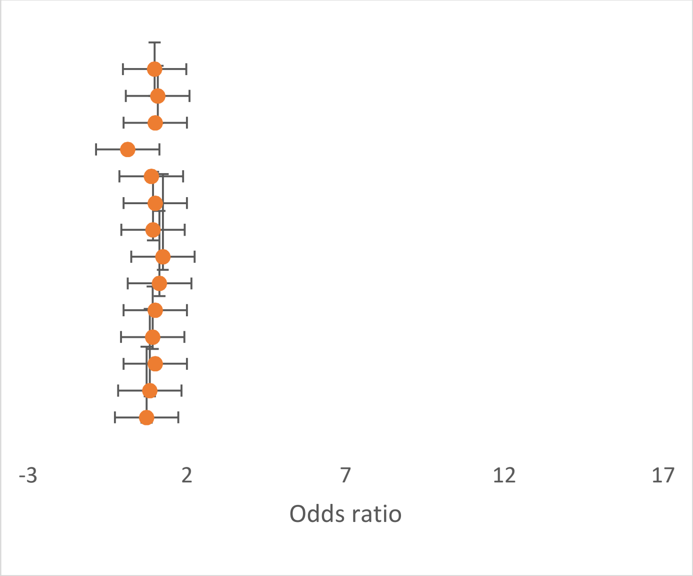
-3
2
7
12
17
Odds ratio

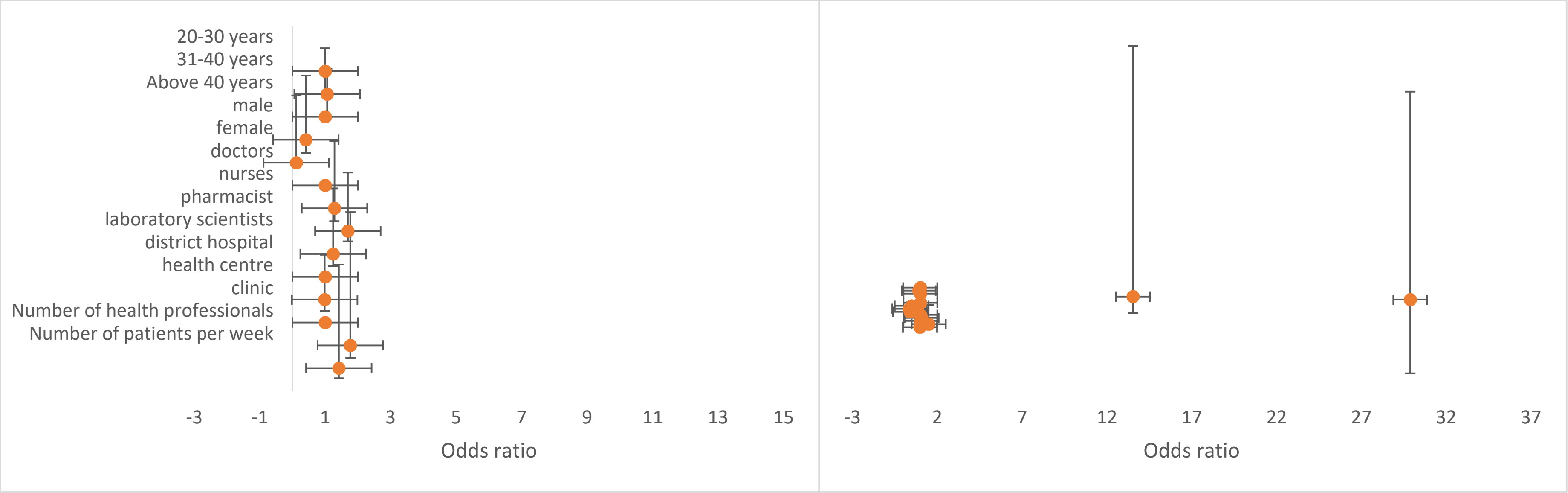

20-30 years
31-40 years
Above 40 years
male
female
doctors
nurses
pharmacist
laboratory scientists
district hospital
health centre
clinic
Number of health professionals
Number of patients per week
-3 -1 1 3 5 7 9 11 13 15
Odds ratio
-3 2 7 12 17 22 27 32 37
Odds ratio

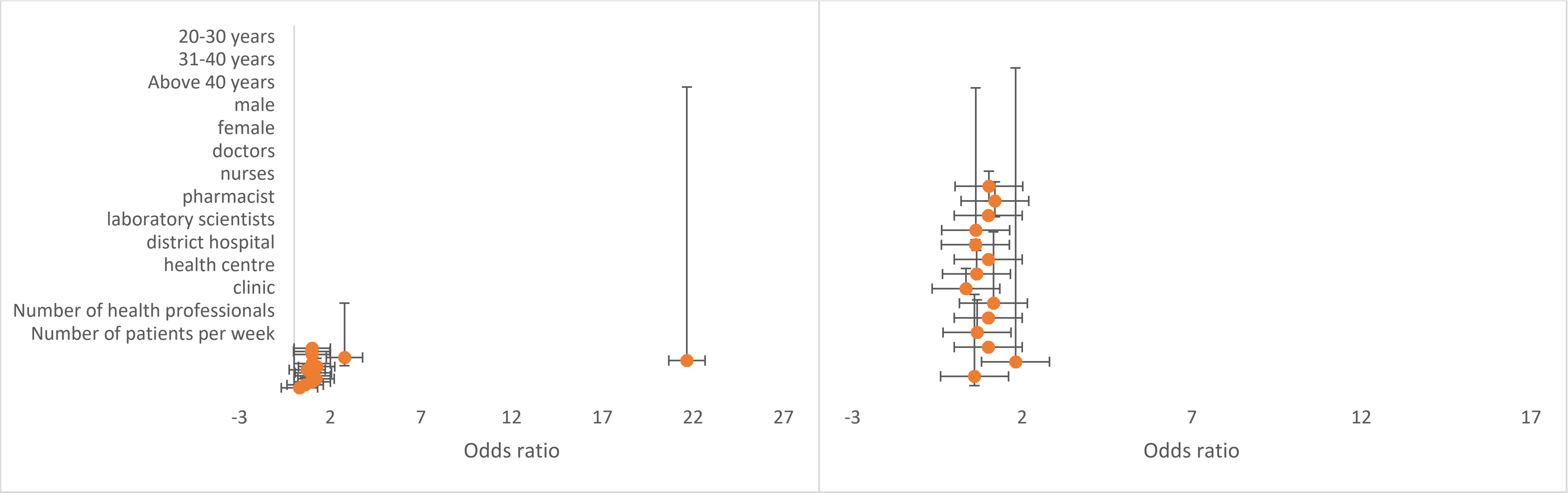

20-30 years
31-40 years
Above 40 years
male
female
doctors
nurses
pharmacist
laboratory scientists
district hospital
health centre
clinic
Number of health professionals
Number of patients per week
-3
2
7
12
17
22
27
Odds ratio
-3
2
7
12
17
Odds ratio

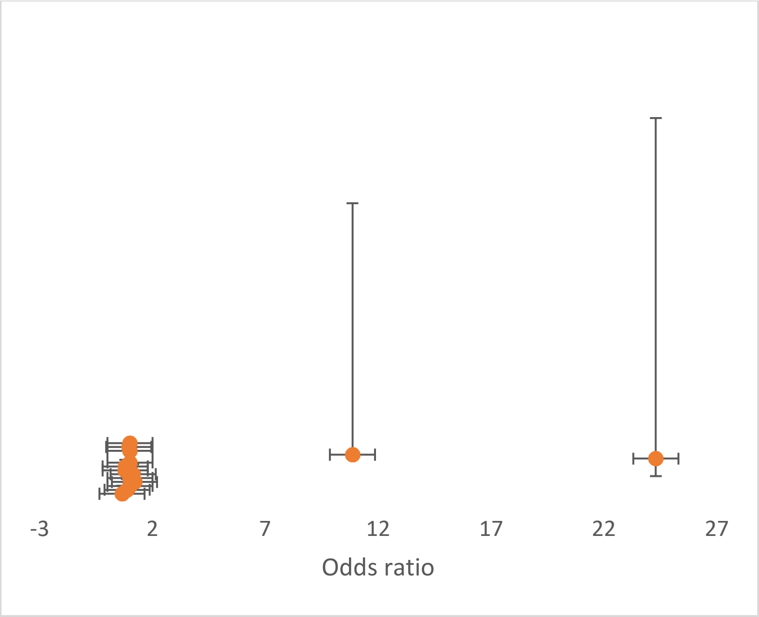

Figure S1. Odds ratio showing the association on the availability of mobile apps, toll-free, supply of power, support systems and others for disease diagnosis and treatment support by health workers in Ashanti Region, Ghana.

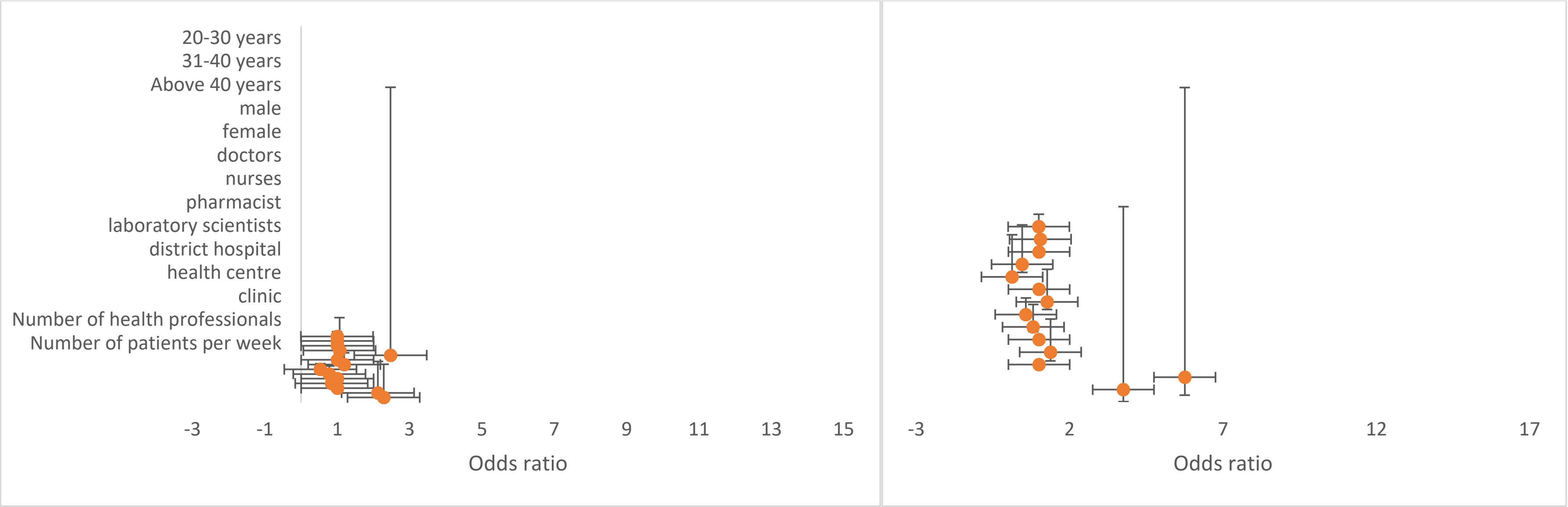
20-30 years
31-40 years
Above 40 years
male
female
doctors
nurses
pharmacist
laboratory scientists
district hospital
health centre
clinic
Number of health professionals
Number of patients per week
-3
-1
1
3
5
7
9
11
13
15
Odds ratio
-3
2
7
12
17
Odds ratio

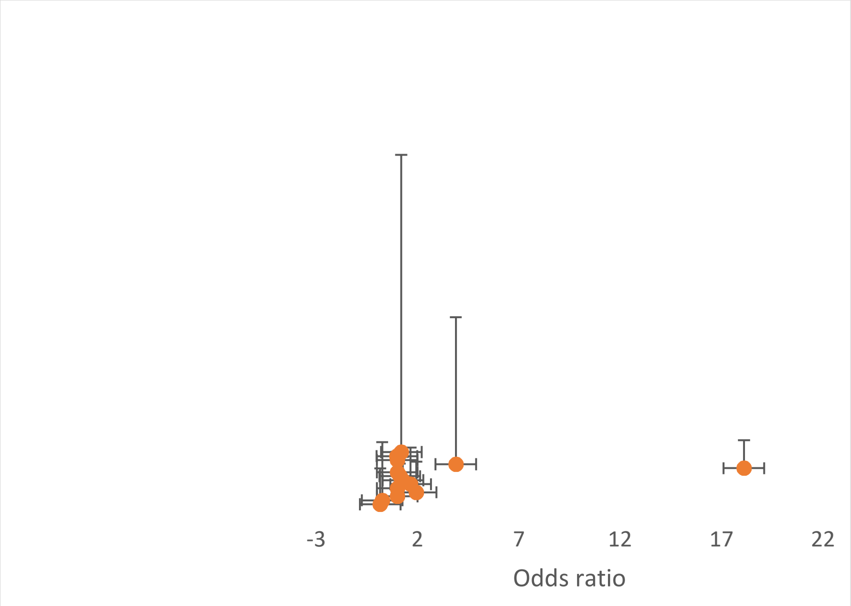
-3
2
7
12
17
22
Odds ratio

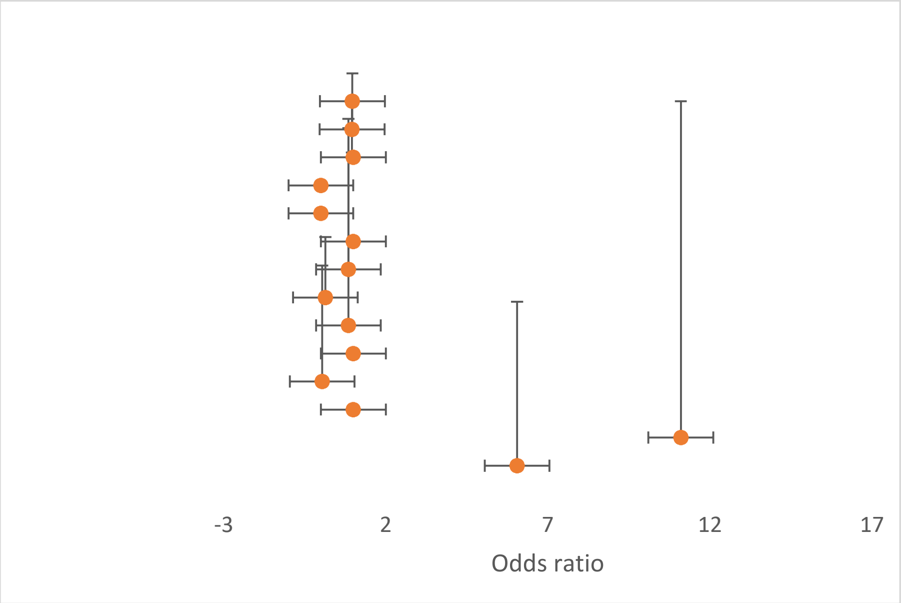
-3
2
7
12
17
Odds ratio

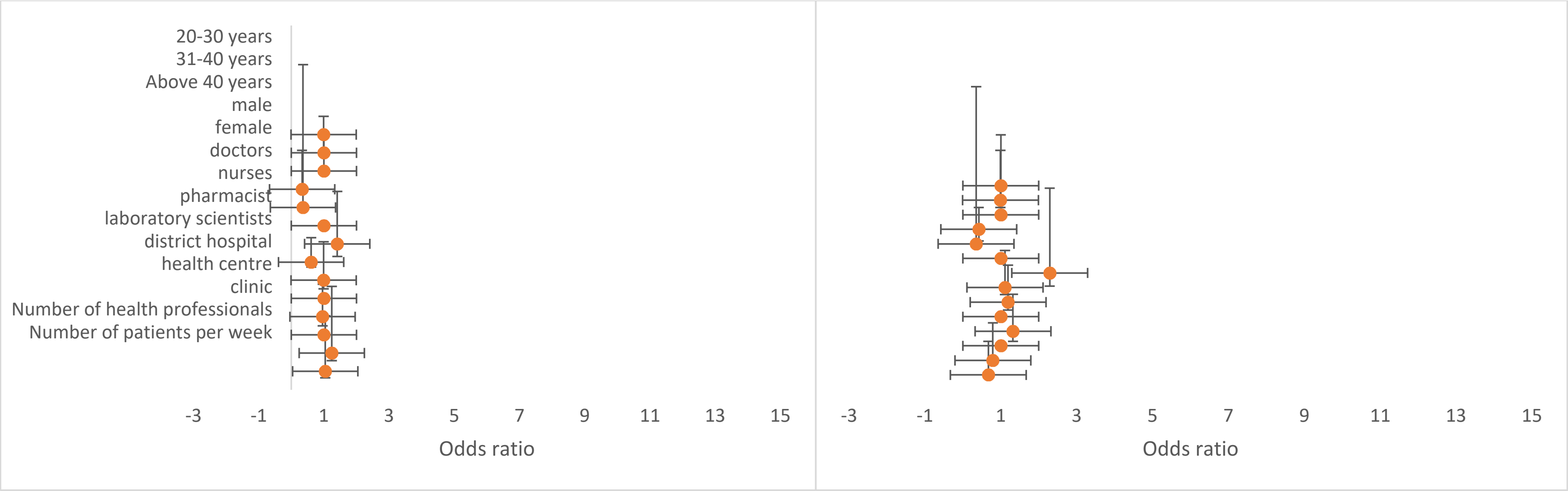

20-30 years
31-40 years
Above 40 years
male
female
doctors
nurses
pharmacist
laboratory scientists
district hospital
health centre
clinic
Number of health professionals
Number of patients per week
-3
-1
1
3
5
7
9
11
13
15
Odds ratio
-3
-1
1
3
5
7
9
11
13
15
Odds ratio

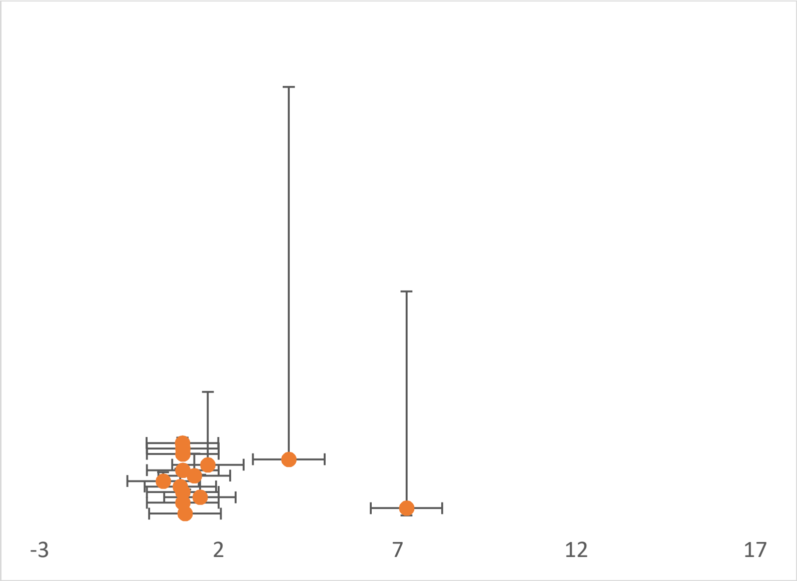
-3
2
7
12
17

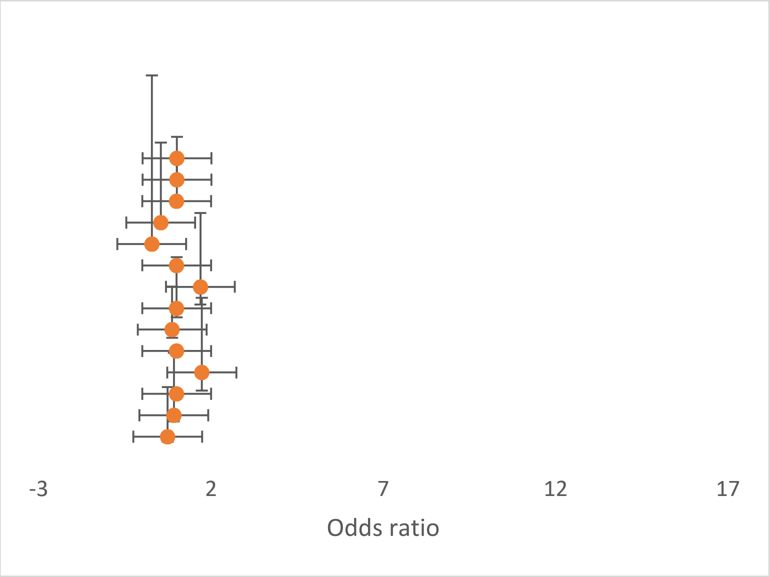
-3
2
7
12
17
Odds ratio

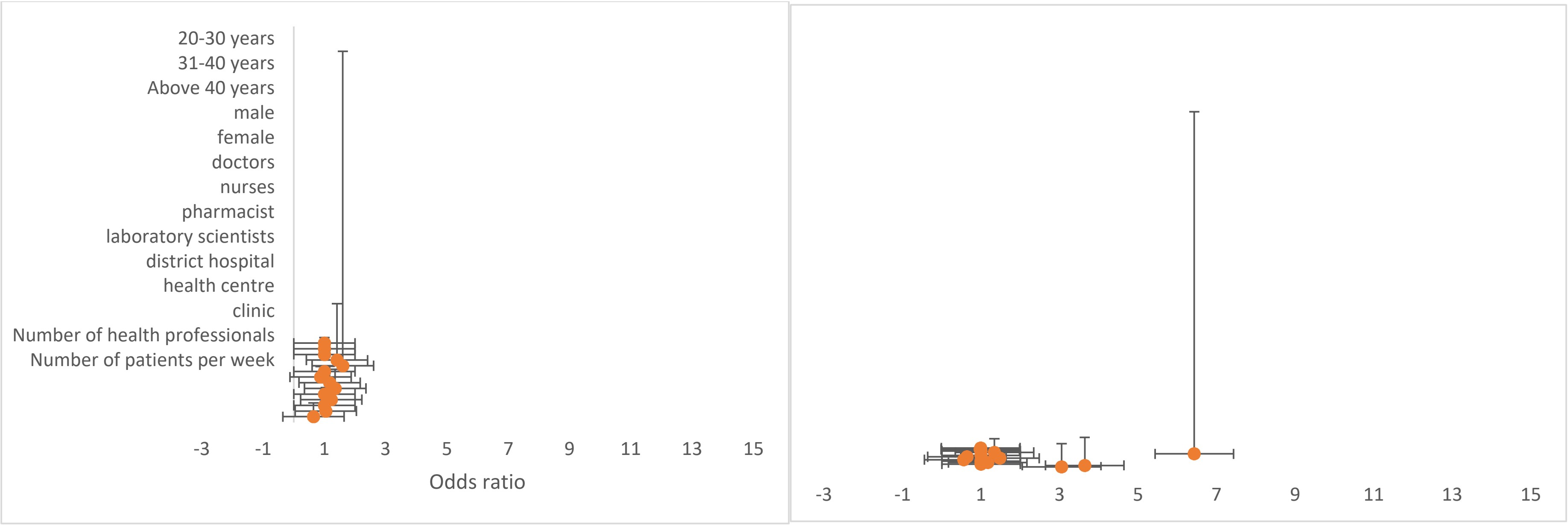

Figure S2. Odds ratio showing the association on the use of mHealth applications for the management and treatment of hypertension, diabetes, cancer, malaria, monitor patients' conditions and others by health workers in Ashanti Region, Ghana.

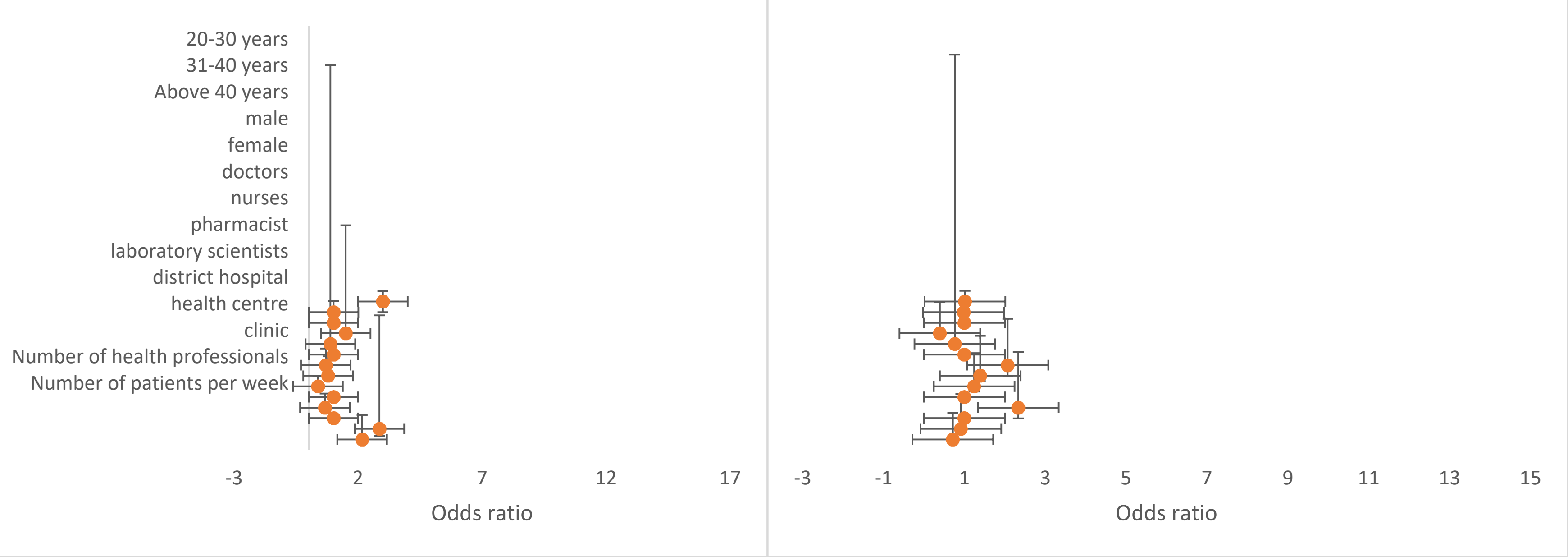

File S1. Distribution of primary healthcare facilities sampled in the Ashanti Region.

**Name of stratum (District) (N=43)**	**Number (N=100)**
Adansi Asokwa	1
Adansi Akrofroum	2
Adansi North	2
Adansi South	2

Afigya Kwabre North	2
Afigya Kwabre South	2
Ahafo Ano North	3
Ahafo Ano South-East	2
Ahafo Ano South-West	2
Amansie Central	2
Amansie South	2
Amansie West	2
Bekwai Municipal	3
Asante Akim Central	2
Asante Akim North	2
Asante Akim South	3
Mampong Municipal	4
Atwima Mponua	2
Atwima Kwawoma	2
Atwima Nwabiagya North	3
Atwima Nwabiagya South	2

Bosomtwe	4
Ejisu Municipal	4
Juaben Municipal	2
Ejura-Sekyredumase	2
Kwabre East	3
Obuasi Municipal	2
Obuasi East	1
Offinso Municipal	2
Offinso North	2
Bosome Freho	2
Sekyere Afram Plains	1
Sekyere Central	2
Sekyere East	2
Sekyere Kumawu	1
Sekyere South	4
Asokore Mampong	2
Asokwa Municipal	1

Kumasi Metro	5
Kwadaso Municipal	2
Old Tafo Municipal	2
Suame Municipal	2
Oforikrom Municipal	3

Survey tool

University of Kwazulu-Natal, Durban, School of Nursing and Public Health, Discipline of Public Health Medicine

Title:* *Mobile health (mHealth) technology for disease diagnosis and treatment support by health professionals in Ghana

Thank you for accepting to participate in this study. This questionnaire has three sections:

Part I: Demographic information

Part II: Availability of mHealth for diagnostics and treatment support

Part III: Use of mHealth for diagnostics and treatment support

Mark with an X in the appropriate box

PART I: DEMOGRAPHIC INFORMATION

Number	Question	Response
1.	Age	20-30 ☐ years<br>31-40 ☐ years<br>41-50 ☐ years<br>51-60 ☐ years
2.	Sex	Male ☐<br>Female ☐
3.	Categories of health professionals	Medical Doctor ☐

		Physician Assistant ☐<br><br>Midwife ☐<br><br>General Nurse ☐<br><br>Community Health Nurse ☐<br><br>Laboratory Scientist/technician ☐<br><br>Pharmacist/Dispensary technicians ☐<br><br>Others (Specify) ________________
4.	Type of facility	☐

		District Hospital<br><br>Sub-district health center ☐<br><br>Clinic ☐<br><br>CHPS ☐
5.	Total number of healthcare professionals	________________________
6.	How many patients do you see per week?	________________________

PART II: AVAILABILITY OF MOBILE HEALTH FOR DIAGNOSTICS AND TREATMENT SUPPORT

Section A: Available Health Infrastructure		
**Number**	**Question**	**Response**
7.	Do you or your facility have mobile wireless devices to support the provision of healthcare?	Yes ☐<br>No ☐
8.	Are there mobile health (mHealth) interventions available in this facility to support healthcare delivery?	Yes ☐<br>No ☐<br>***If No (Go to Section D)***

9.	What are the various types of mobile health interventions available to health professionals in this facility?<br><br>*Tick all that apply*	Text message ☐<br><br>Voice/phone calls ☐<br><br>Mobile apps ☐<br><br>Multimedia messaging ☐<br><br>Video conferencing ☐<br><br>Emergency toll-free lines ☐<br><br>Others (Specify) ___________<br><br>___________

10.	What are the various types of wireless devices available to health professionals for mobile health interventions in this facility?<br><br>*Tick all that apply*	Mobile phones ☐<br><br>Smartphones ☐<br><br>Tablets ☐<br><br>Personal digital assistants ☐<br><br>Handheld devices ☐<br><br>Patient monitoring devices ☐<br><br>Watches ☐
11.	Do you have continuous supply of power supply to support mobile health interventions in this facility?	Yes ☐<br><br>☐

		No
12.	Are there support systems available for the existence of mHealth for diagnostics and treatment support?	Yes ☐<br><br>No ☐<br><br>***If Yes list them*** ____________<br><br>____________________<br><br>____________________<br><br>____________________<br><br>____________________

	Section B: Healthcare Workforce Competency	
13.	Do you have the requisite skills to use mHealth interventions for diagnostic purposes?	Yes ☐<br><br>No ☐<br><br>**If Yes list the skills** ________<br><br>________________________<br><br>________________________<br><br>________________________

		______________________
14.	Do you have the competence to use mHealth interventions to treat, monitor and manage diseases?	Yes ☐<br>No ☐

PART III: USE OF MOBILE HEALTH FOR DIAGNOSTICS AND TREATMENT SUPPORT

Section C: You and Your Mobile Wireless Device		
15.	Have you ever used or currently using mHealth interventions to support healthcare delivery?	Yes ☐<br>No ☐

16.	What type of disease(s) has mHealth been used or is currently being used for in this facility?<br><br>*Tick all that apply.*	HIV ☐<br><br>TB ☐<br><br>Hypertension ☐<br><br>Diabetes ☐<br><br>Stroke ☐<br><br>Cancer ☐<br><br>Chronic Respiratory disease ☐<br><br>Malaria ☐<br><br>Diarrhoea ☐

		Others (Specify) _____________<br><br>_____________
17.	Have you ever used smart mobile wireless device to:<br><br>*Tick all that apply.*	Find health or medical information ☐<br><br>Disease diagnosis ☐<br><br>Treat and manage disease conditions ☐<br><br>Treat and monitor patients' health conditions ☐
18.	How often do you use mobile wireless device for diagnostic purposes?	Once a month ☐

		2 or 3 times a month ☐<br>1 to 6 times a week ☐<br>Once a day or more ☐
19.	How often do you use mobile wireless device for treatment, monitoring and management of diseases?	Once a month ☐<br>2 or 3 times a month ☐<br>1 to 6 times a week ☐<br>Once a day or more ☐
Section D: Usefulness of mobile health interventions		

20.	Do health professionals use mHealth to monitor patients' disease conditions?	Yes ☐<br>No ☐
21.	Do health professionals use mHealth to manage non-communicable diseases like diabetes, hypertension etc?	Yes ☐<br>No ☐
22.	Do health professionals use mHealth to manage communicable diseases such as HIV, TB etc?	Yes ☐<br>No ☐

23.	Do health professionals use mHealth as reminders to improve their treatment adherence procedures?	Yes	☐
		No	☐
24.	Do health professionals use mHealth as reminders to promote patients' medication adherence?	Yes	☐
		No	☐
25.	Do health professionals use mHealth to remind patients to honour their clinic appointments?	Yes	☐
		No	☐

26.	Do health professionals use mHealth to remind patients to collect their ART and other drugs on time?	Yes ☐<br>No ☐
27.	Do health professionals use mHealth for follow-ups to promote treatment compliance?	Yes ☐<br>No ☐
28.	Do health professionals use mHealth to support patients test result notifications?	Yes ☐<br>No ☐

29.	Does the use of mHealth improve the treatment and management of disease conditions?	Yes ☐<br><br>No ☐
30.	Do health professionals use mHealth for making accurate diagnostic decisions?	Yes ☐<br><br>No ☐
31.	Does the use of mHealth intervention increase the effectiveness of treatment and management of diseases?	Yes ☐

		No ☐
	Section E: Ease of Use	
32.	Is it easy to use mobile health interventions to support disease diagnosis?	Yes ☐<br>No ☐
33.	Is it easy to use mobile health interventions to support the treatment of patients' disease conditions?	Yes ☐<br>No ☐

34.	Is it flexible to interact with mobile health devices for disease diagnosis and treatment support?	Yes ☐<br>No ☐
35.	Is it frustrating to interact with mobile health devices for disease diagnosis and treatment support?	Yes ☐<br>No ☐
36.	Is it easy for me to become skilful in using mHealth for disease diagnosis and treatment support?	Yes ☐<br>No ☐

37.	Is it easy for me to learn how to use mobile health devices for diagnosis and treatment support would be easy for me?	Yes ☐<br>No ☐
	Section F: User Satisfaction	
38.	Do you feel comfortable in using mobile health for disease diagnosis and treatment procedures?	Yes ☐<br>No ☐

39.	Are you confident in using mobile health for disease diagnosis and treatment procedures?	Yes ☐<br>No ☐
40.	Are you completely satisfied in using mobile health for disease diagnosis and treatment procedures?	Yes ☐<br>No ☐
41.	Do you believe that using mHealth for disease diagnosis and treatment support will increase the quality of healthcare delivery?	Yes ☐<br>No ☐

Section G: Behavioural Intention to Use		
42.	Would you use mHealth for the treatment and management of patients' disease conditions?	Yes ☐<br>No ☐
43.	Would you as a health professional, always use mHealth for disease diagnosis and treatment support?	Yes ☐<br>No ☐

44.	If you have access to mHealth, do you intend to use it for disease diagnosis and treatment support?	Yes ☐<br>No ☐

Thank you for your cooperation
